# Combined System of Magnetic Resonance Sounding and Time-Domain Electromagnetic Method for Water-Induced Disaster Detection in Tunnels

**DOI:** 10.3390/s18103508

**Published:** 2018-10-17

**Authors:** Xinlei Shang, Chuandong Jiang, Zhongjun Ma, Shengwu Qin

**Affiliations:** 1 College of Instrumentation and Electrical Engineering, Jilin University, Changchun 130061, China; shangxinlei@jlu.edu.cn (X.S.); chuandongjiang@gmail.com (C.J.); 2 College of Construction Engineering, Jilin University, Changchun 130061, China; mzjgeology@gmail.com

**Keywords:** instrument, magnetic resonance sounding, time-domain electromagnetic, water, tunnel

## Abstract

Underground construction projects such as tunnel construction are at high risk of water-induced disasters. Because this type of disaster poses a serious threat to worker safety and productivity, instruments and methods that can accurately detect the water source are critical. In this study, a water detection instrument that combines Magnetic Resonance Sounding (MRS) and Time-domain Electromagnetic Method (TEM) techniques to yield a joint MRS-TEM interpretation method was developed for narrow underground spaces such as tunnels. Joint modules including a transmitter and receiver were developed based on a dual-purpose and modular design concept to minimize the size and weight of the instrument and consequently facilitate transportation and measurement. Additionally, wireless control and communication technology was implemented to enable inter-module cooperation and simplify instrument wiring, and wireless synchronization was accomplished by implementing a Global Positioning System (GPS)-based timing scheme. The effectiveness and reliability of the instrument were verified via indoor laboratory tests and field measurement signal tests. Furthermore, the practicability of the combined instrument and its interpretation method was verified via a field case performed in a tunnel in Hubei, China.

## 1. Introduction

With rapid socio-economic development and changes in the global climate, the world is facing the reality of increasingly urgent water resource problems. However, uncontrolled water sources can also negatively impact human production and endanger the lives of construction workers. In particular, during underground construction work, such as tunneling or mining, heavy water and mud inrush can seriously injure the construction workers and impede the development of the construction project [1,2]. Thus, in order to detect water sources quickly and accurately so as to ensure the safe construction of tunnels, many domestic and international experts and scholars have systematically investigated advanced detection methods for tunnels.

The time-domain electromagnetic method (TEM) has several advantages such as a wide range of depth of detection, rich information and high efficiency [3]. However, the resistivity results obtained via the TEM tend to be anomalous, and the cause for this cannot be directly identified as water. In addition, the method does not provide a quantitative analysis of the hydrological characteristics. On the contrary, Magnetic Resonance Sounding (MRS), as a direct and quantitative method to detect groundwater sources, has the advantages of high resolution, high efficiency, rich information and unique interpretation [4]. However, accurate interpretation of the MRS method requires resistivity to be a priori information [5]. Therefore, combining the MRS and TEM methods would enable selective application of the desirable features of both methods, while also suppressing the undesirable features, thereby effectively improving the detection accuracy of water bodies in tunnels.

The TEM and MRS method both require resistivity information for application. Specifically, MRS modeling requires knowing the resistivity, as it not only affects the amplitude, but also the phase of the MRS signal [6], and applying the assumption of a uniform half-space resistivity will result in inaccurate inversion results [5]. In their study, [7] attempted to invert the resistivity directly from the amplitude and phase of the MRS signal. However, in practical applications, the influence of environmental noise on the phase data causes the resistivity results to be inaccurate. The work in [8] proposed an inversion method that combines MRS and the TEM and implemented it under complex geological conditions; consequently, the results confirmed the ability of the method to locate aquifer locations accurately. Subsequently, [9] investigated the Lateral Constrained Inversion (LCI) method and applied it to the combined constrained inversion of MRS data, terrestrial TEM data and aerial TEM data; through their method and application, they were able to improve the accuracy of the inversion interpretation results significantly. The work in [10] proposed adding the Adaptive Genetic Algorithm (AGA) to the combined inversion achieved via MRS and TEM implementation in order to determine the real-time groundwater content from the resistivity distribution information. The effectiveness and practicability of their combined method were verified via their results.

Although combining MRS and the TEM yields significant advantages, their measurement processes are independently performed. Commercial MRS instruments, such as GMR (Vista Clara, Santa Clara, CA, USA) and NUMIS (Iris, Orléans, France), are mainly employed for ground applications [11,12] and are often large in size and weight. In recent years, MRS instruments purposed for tunnels and mines have been substantially reduced in size and weight so that they can be used in narrow space environments [13,14,15]. Similarly, the most commonly-employed commercial TEM instruments available for underground measurements are the EM-47 (Geonics Limited, Mississauga, ON, Canada) and V8 (Phoenix Geophysics, Scarborough, ON, Canada); these instruments not only have the same problem in terms of size and weight, but they also have limited functionality.Considering this, [16] developed a combined MRS-TEM instrument based on the similarities between MRS and TEM instruments; consequently, test results demonstrated that their proposed instrument could effectively perform groundwater detection. In this study, a combined MRS-TEM instrument with a reduced size and weight was developed for application in a narrow underground environment. Because the proposed instrument design is based on the idea of an integrated system, the proposed instrument is able to integrate the features and principles of the two methods and achieve better performance and increased applicability in tunnels and mines.

This paper describes the basic principles of the TEM and MRS method and their joint inversion process, with emphases on descriptions of key technologies, including the structure of the combined MRS-TEM instrument, wireless control transmission, Global Positioning System (GPS) synchronization, transmission timing control and signal processing. Then, the indoor simulation tests that were performed on the major modules of the combined instrument are described and discussed. Finally, field tests that were conducted in a tunnel near Qiyue Mountain in Hubei Province, China, to verify the effectiveness of the proposed instrument are described, and the MRS-TEM combined advanced detection results are presented and discussed.

## 2. Combined Detection Method

### 2.1. MRS Method

**Figure 1:**
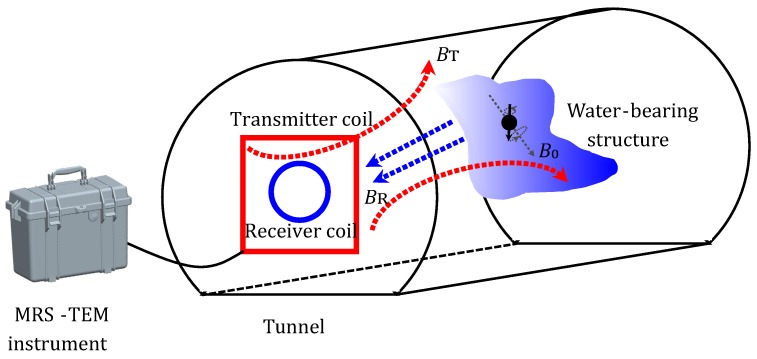
Illustration of the combined Magnetic Resonance Sounding (MRS) and Time-domain Electromagnetic Method (TEM) detection method, using advanced tunnel detection as an example.

When implementing the MRS method, the geomagnetic field $B_{0}$ is considered to be the background magnetic field, which has a corresponding Larmor (angular) frequency of $\omega_{L}=\left|\gamma B_{0}\right|$, where γ is the gyromagnetic ratio of the hydrogen proton, as is shown in Figure 1. The hydrogen proton spin produces a macroscopic magnetization *M*, which is also referred to as the net magnetization vector $M_{0}$ at equilibrium [17]; its direction is the same as that of $B_{0}$. An alternating current with the Larmor frequency passing through a transmitter coil generates a magnetic field $B_{1}$ throughout the subsurface that causes *M* to tilt towards $B_{1}$. However, when this alternating current is switched off, *M* gradually relaxes and realigns with $B_{0}$; as this occurs, a free induction decay signal is generated in the ground coil. This signal, as is described below, is the MRS signal [4]:

(1) $$V\left(t\right)=e_{0}\left(q,\rho \right)e^{-t/T_{2}^{*}}\cos\left(2\pi f_{L}t+\phi \left(q,\rho \right)\right)$$

where $q=I_{0}\tau_{p}$, i.e., the product of the transmission current intensity and transmission time, is the pulse moment; $e_{0}$ is the initial amplitude of the signal, which is directly proportional to water content; $T_{2}^{*}$ is the relaxation time and is related to the pore size; $f_{L}$ is the Larmor frequency; ϕ is the phase of the signal, which is related to the underground conductivity ρ.

Suppose a unit volume r in the subsurface space has a unique water content *w* (0≤w≤1) and a unique relaxation time $T_{2}^{*}$; then, the initial amplitude of the MRS signal received by the receiver coil, $e_{0}$, can be expressed as follows [18]:

(2) $$e_{0}\left(q,\rho \right)=\int K\left(q,\rho ,r\right)w\left(r\right)d^{3}r$$

where *K* is the kernel function and describes the sensitivity of the MRS signal to $w\left(r\right)$; *K* is dependent on the measurement environment (i.e., the strength of $B_{0}$, geomagnetic inclinations *D* and *I* and environmental temperature *K*), subsurface resistivity ρ and parameters such as the coil geometry and instrumental measurements [6,19] (Weichman et al., 2000; Hertrich et al., 2009). Refer to [20] or [18] for a more detailed explanation of MRS.

### 2.2. TEM Method

When the TEM is implemented, a large fixed-source loop and center loop are often employed for detection. Under these conditions, an abrupt switch-off of a bipolar current passing through a transmitter coil generates the primary magnetic field, which is a magnetic field that surrounds the transmission loop line. During transmission, if the primary magnetic field encounters a good subsurface conductor, a current is induced within the conductor, which then generates a surrounding secondary magnetic field [21,22]. However, heat loss of the induced current in the good conductor causes this secondary magnetic field to decay exponentially. The decay of the induced subsurface secondary magnetic field over time is monitored and quantified by a receiver coil; these results are then implemented in the inversion calculation to obtain the underground resistivity distribution information. Using an underground vertical magnetic field as an example, the TEM time domain response of the center loop can be expressed as:

(3) $$V_{z}=\int_{-\inf}^{\inf}\frac{Ia}{2\pi }\int_{0}^{\inf}\frac{\lambda^{2}}{\lambda +u_{1}\left(\rho \right)}e^{-u_{1}\left(\rho \right)z}J_{1}\left(\lambda a\right)d\lambda e^{-i\omega t}d\omega$$

where *I* is the transmission current; *a* is the radius of the transmitter coil; $u_{1}$ is the effective wavenumber determined from the distribution of subsurface resistivity ρ; $J_{0}$ and $J_{1}$ are the zero-order and first-order Bessel functions, respectively [23]. Note that Equation (3) contains an integral Bessel function. However, as a result of its oscillatory nature, the Bessel function cannot be analytically expressed. Thus, the forward process of the TEM is actually the process of solving the double integral Bessel function [23,24].

### 2.3. Combined Interpretation

It can be seen from Equations (1)–(3) that the underground resistivity distribution ρ is taken into account in both the MRS method and TEM. Although the distribution of ρ can be derived by inverting the TEM data, the water content distribution cannot be directly determined. In contrast, the MRS method requires an accurate distribution of ρ to establish the kernel function and then to obtain the water content distribution. Therefore, through the joint inversion of these two methods, their respective limitations can be overcome, and the distributions of ρ and water content can be obtained.

**Figure 2:**
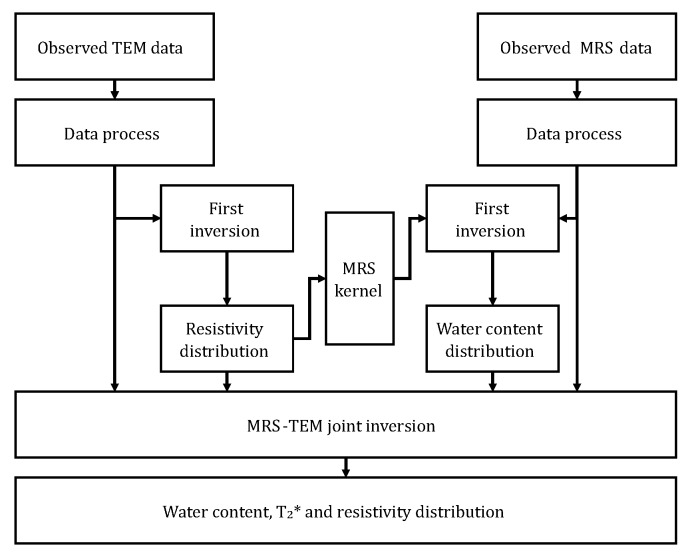
Illustration of the combined MRS-TEM interpretation process.

The process of combined MRS-TEM inversion is illustrated in Figure 2. First, after the noise canceling data collected via the TEM are processed, an independent TEM inversion is performed, and the distribution of ρ is subsequently derived. This distribution is then used to calculate the kernel function *K* of the MRS. Then, after noise canceling, data collected via the MRS method are processed; an independent MRS inversion is performed; and the distributions of the preliminary water content and $T_{2}^{*}$ are derived. Finally, the distributions of ρ, water content and $T_{2}^{*}$ are used as the initial values to perform an MRS-TEM joint inversion. The target function of the joint inversion is given as [10]:

(4) $$\Phi =w_{\mathrm{TEM}}∥D_{\mathrm{TEM}}\left[V_{\mathrm{TEM}}^{\mathrm{obs}}-V_{\mathrm{TEM}}^{\mathrm{calc}}\left(\rho \right)\right]{∥}_{2}^{2}+w_{\mathrm{MRS}}∥D_{\mathrm{MRS}}\left[V_{\mathrm{MRS}}^{\mathrm{obs}}-V_{\mathrm{MRS}}^{\mathrm{calc}}\left(\rho \right)\right]{∥}_{2}^{2}+\lambda {∥\mathrm{Cm}\left(w,T_{2}^{*},\rho \right)∥}_{2}^{2}$$

where $w_{\mathrm{TEM}}$ and $w_{\mathrm{MRS}}$ are the weighting factors; $V_{\mathrm{TEM}}^{\mathrm{obs}}$ and $V_{\mathrm{MRS}}^{\mathrm{obs}}$ are the acquired observation data; $V_{\mathrm{TEM}}^{\mathrm{calc}}$ and $V_{\mathrm{MRS}}^{\mathrm{calc}}$ are the forward processing data calculated via MRS and the TEM; C is the smoothness matrix; and m is the variable matrix containing ρ, *w* and $T_{2}^{*}$. λ is the regularization parameter that is used to normalize data fitting and variable smoothness [25]. By using the conjugate gradient algorithm [26] to iteratively solve Equation (4) and by automatically adjusting the regularization parameters at each iteration, the underground distributions of *w*, $T_{2}^{*}$ and ρ can be derived.

## 3. Combined MRS-TEM Instrument

### 3.1. Instrument Frame

**Figure 3:**
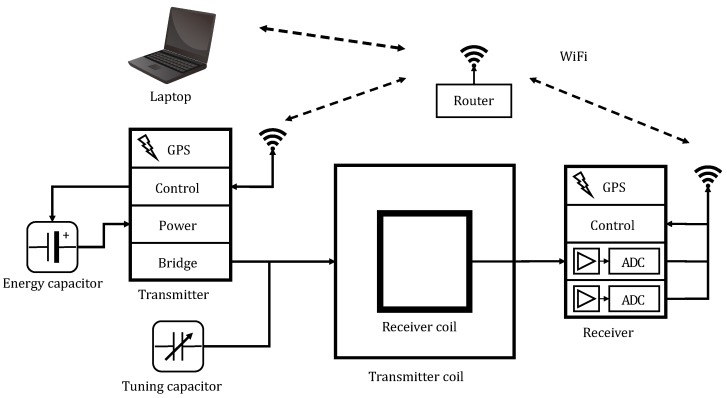
Functionality diagram of the combined MRS-TEM instrument.

The combined MRS-TEM instrument has two working modes that are respectively based on MRS and the TEM. Its primary components include a laptop, a transmitter, a receiver, a power module, tuning capacitors and transmitter and receiver coils, as is shown in Figure 3. The laptop is equipped with software that controls the instrument, processes and displays measured signals and saves measured data. The transmitter includes modules for transmitter control and a transmitter bridge that collaborate with the power module, tuning capacitors and the transmitter coil to transmit the MRS- or TEM-derived current. The receiver includes modules for signal tuning and processing; it also includes an analog-to-digital conversion module that amplifies and filters the acquired MRS or TEM signal and converts it into digital values. The power module, which is connected to the battery to supply power to the combined instrument, is comprised of energy capacitors and a step-up control module.

Control signal communication and data transmission between the laptop, transmitter and receiver were enabled by WiFi. In an open space, WiFi transmission is possible for a distance of up to 200 m, and the data are transmitted within a frequency range of 2.4–5 GHz in order to prevent signal interference. The relationship between the laptop and each module follows the master/slave protocol and utilizes the client/server structure, with the laptop being assigned the role of client. The slaves include the transmitter control module, the receiver control module and the receiver capture card, which are all servers and are connected to a wireless router that operates in the Access Point (AP) mode.

When the combined MRS-TEM instrument is performing TEM-based measurements, there are two synchronization methods: line synchronization and GPS synchronization. Under the condition that the distance between the transmitter and receiver is relatively large, the GPS synchronization method is more practical than the line synchronization method. This is because the GPS synchronous timing module (LEA-6T) simultaneously outputs two pulse signals with a pulse frequency of up to 10 MHz and synchronization pulse error of less than 30 ns. One of the pulse signals is used as the clock reference for the transmitter and receiver control modules, whereas the other pulse signal is used as the reference pulse to synchronize the start of operation. However, when the transmitter and receiver are close together, such as in tunnel applications, the line synchronization method is more convenient and reliable.

### 3.2. Transmitter

The transmitter module comprises a transmitter control module, power control module, transmitter bridge module and current collection module. The transmitter employs an ARM Micro-Controller Unit (MCU) as its core and performs the following five functions:

1. Data communication: The transmitter communicates with the laptop via serial ports and Ethernet conversion circuits.
2. Transmitter control parameter calculation: The transmitter transmits analog SPI to a Complex Programmable Logic Device (CPLD) via a digital IO port to generate the MRS-based and TEM-based transmission timing signals.
3. MRS power control: The transmitter is capable of increasing or decreasing the output voltage according to the commands from the laptop.
4. TEM transmission switch-off current collection: The transmitter can achieve current-data acquisition with 12-bit accuracy, an 800-Kps sample rate and a 1-ms collection time via an integrated Analog-to-Digital converter (ADC) in the MCU.
5. GPS unit configuration: The transmitter acquires GPS data via serial ports.

The transmitter timing control parameters include the TEM transmission frequency division factor, MRS transmission frequency division factor and MRS state machine time point count. In addition, the transmission mode can be switched between the MRS and TEM mode; this is achieved by exporting the high and low levels to the CPLD via an IO port of the MCU. The clock of the CPLD is a 20-MHz active crystal oscillator that divides the frequency according to the frequency division factor to derive the respective transmission frequency required by the MRS or TEM mode. As an example, $m_{\mathrm{MRS}}=20\times 10^{6}\;\mathrm{Hz}/f_{0}$, where $m_{\mathrm{MRS}}$ is the division factor and $f_{0}$ is the transmission frequency for the MRS mode. The MRS detection cycle consists of three stages. During the first stage, noise is collected, but no current is transmitted; then, in the second and third stages, complete transmission and collection processes are performed. The state machine is used to describe states such as idle, transmit, collect and wait and to calculate the duration of each state.

Because the IO ports of the MCU and CPLD are both TTL ports with a limited current output capacity, a driver circuit is used to enhance the driving ability of the control signal. The driver circuit is made with a Darlington array chip (ULN2803AG) and a MOSFET integrated chip (AO4606). An ULN2803AG chip is used to drive the power control signal, ADC control signal and control signal for the MRS signal-conditioning module. The AO4606 chip is used to drive the MRS- and TEM-based bridge control signals. Once a step-up command is received, the power control module collects the voltage of the energy capacitors via the ADC built into the MCU. If the voltage is lower than the requested step-up value, the module issues a step-up control signal to the power control unit; otherwise, a step-down control signal is issued.

### 3.3. Receiver

As previously mentioned, the receiver is comprised of a receiver control module, a signal processing module and an analog-to-digital conversion module. The receiver implements the MCU and CPLD as its core and is capable of functions such as serial-to-Ethernet conversion, receiver control timing generation and GPS synchronization. The MRS signal processing module includes a high-voltage relay, a tuning circuit, a preamplifier, high-pass and low-pass filters and a final amplifier [13]. The high-voltage relay prevents high voltage from damaging the receiver during the MRS transmission phase.

The capacitors and resistances in the tuning circuit and receiver coil form an LCRtuning circuit that is purposed to select the frequency of the received signal. The preamplifier has a low noise coefficient and performs preliminary amplification of the received signal. The high-pass and low-pass filters are used to reduce the ambient noise outside of the predetermined frequency band. In the final step, the received signal is amplified by the final amplifier before it is transmitted to the ADC. Conversely, the TEM signal processing circuit is relatively simple, as the received signal only passes through a low-pass filter (10 kHz bandwidth) and an amplifier before being transmitted to the ADC. The ADC is a 24-bit, 50-kHz high-speed capture card that is capable of high-speed data communication with the laptop via WiFi.

**Figure 4:**
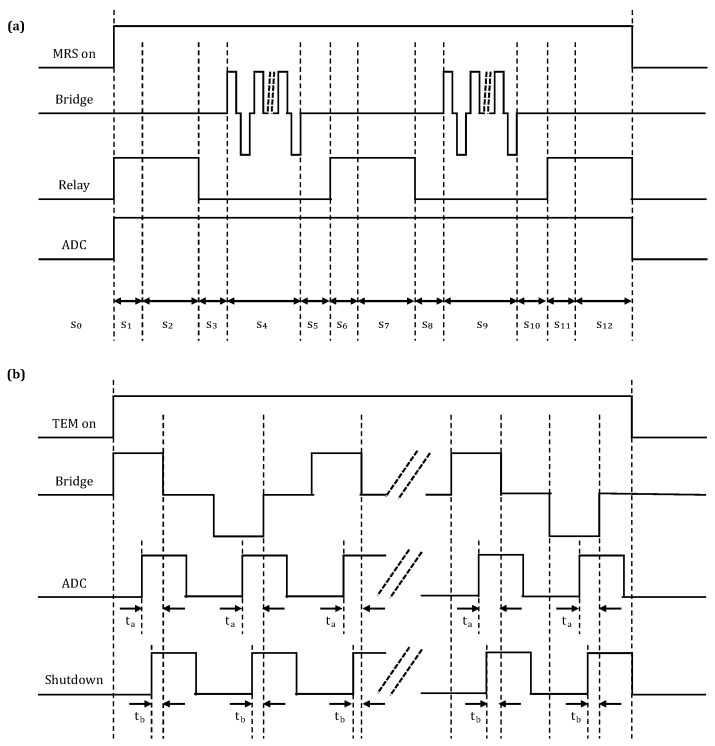
MRS and TEM transmitter and receiver control signal timing scheme. (**a**) MRS timing scheme; (**b**) TEM timing scheme. s, stage.

The control timing scheme for the MRS receiver is illustrated in Figure 4a; as is shown, it has 12 stages: idle s0, relay-operating s1, collect s2, wait s3, transmit s4, wait s5, relay-operating s6, collect s7, wait s8, transmit S9, wait s10, relay-operating s11 and collect s12. The control timing scheme for the TEM receiver in the GPS synchronization mode is shown in Figure 4b. When the start-up signals from the MCU and TEM mode signal become valid, the system enters the pre-start state. Then, when the rising edge of the synchronous start-up signal from the GPS module is captured, the analog-to-digital conversion module control signal is activated. As this occurs, the CPLD in the transmitter bridge control module issues a signal to trigger the collection of the switch-off current.

## 4. Combined System Test

### 4.1. MRS Function Test

#### 4.1.1. Control Timing Test

**Figure 5:**
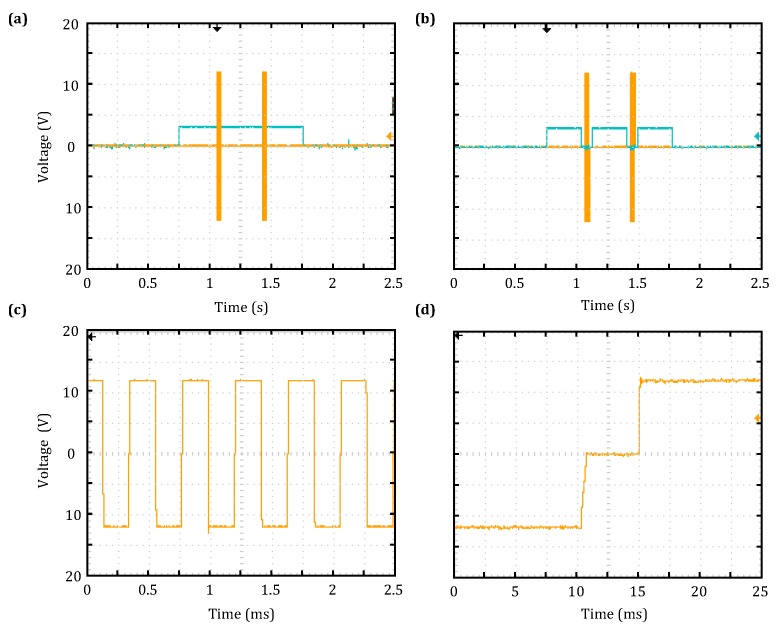
MRS transmitter and receiver control timing test results. (**a**) Transmitter control signal and data collection signal; (**b**) transmitter and relay control signals; (**c**) a portion of the transmitter control signal; (**d**) interval between transmitter bridge circuit control signals.

During the test, the MRS transmission frequency was set to 2326 Hz by the host computer. The waveforms of the MRS transmitter and receiver control signals measured by the oscilloscope are shown in Figure 5. The yellow lines in Figure 5a,b are the transmitter control signals, which correspond to the two transmission currents at s4 and s9 in Figure 4. The blue line in Figure 5a is the ADC control signal, which is collected throughout the duration of the test. The blue line in Figure 5b is the high-voltage relay control signal.

In a single measurement cycle, the relay control signal has three stages of high-level output (i.e., the relay is closed), which are noise collection, and the first and second rounds of signal collection, corresponding to s2, s7 and s12 in Figure 4, respectively. By using the oscilloscope results shown in the screenshots of Figure 5c,d, the transmission frequency was determined to be 2326 Hz, and the interval between bridge control signals was 9.6 μs; both of these values are consistent with the set values.

#### 4.1.2. Receiver Signal Test

An MRS signal collection test was performed at a field experiment site in Changchun, China, where the implemented transmitter and receiver coils were both 100 m × 100 m single-turn square coils. The transmission frequency was 2326 Hz, which was consistent with the local Larmor frequency; the transmission current and corresponding pulse moment were 50 A and 2 As. The bandwidth of the receiving narrowband filter circuit was 100 Hz.

**Figure 6:**
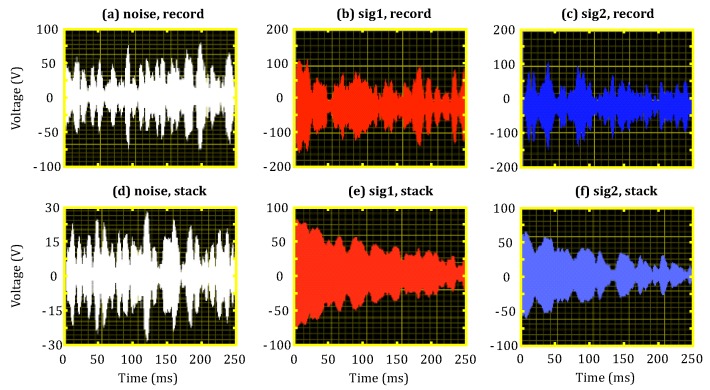
MRS receiver test results. (**a**) Individual noise record; (**b**) individual result of first signal record; (**c**) individual result of second signal record; (**d**) stacked noise record; (**e**) stacked result of first signal record; (**f**) stacked result of second signal record.

The single-measurement and stacked results following 16 repeated measurements are shown in Figure 6. Figure 6a,d illustrates the noise data collected at the s2 stage; the noise levels of the individual and stacked data were 30 nV and 7.5 nV, respectively. Figure 6b,e shows the first round of signal data collection from the s7 stage; although only a blurred MRS signal can be observed from the individual result, the stacked result yields a clear MRS signal. Figure 6c,f illustrate the second round of signal data collection from the s12 stage. It can be seen that the stacked result from the second round of collected signal data also yields clear MRS signals. Because of the different transmission current intensities, the initial amplitudes of the MRS signals collected for the first and second rounds were 75 nV and 60 nV, respectively. Conversely, the relaxation time $T_{2}^{*}$ was 210 ms for both batches of collected signal data.

### 4.2. TEM Functionality Test

#### 4.2.1. Control Timing Test

**Figure 7:**
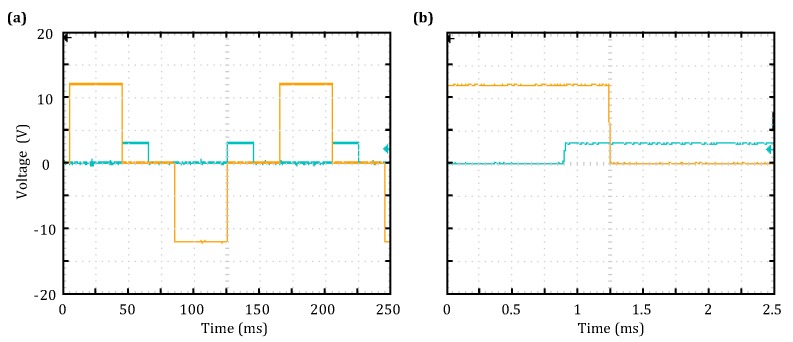
TEM transmitter and receiver control timing test results. (**a**) Full time span of voltage measurement; (**b**) an enlarged view of voltage measurement results corresponding to transmitter bridge switch-off.

During the test, the TEM transmission frequency was set as 6.25 Hz by the host computer. The TEM transmitter and receiver control signals measured when the system was in the GPS synchronization mode are shown in Figure 7. The yellow and blue lines in Figure 7a are the TEM transmitter bridge control signal and trigger signal for switch-off current data collection, respectively; the results were found to be in agreement with the designed timing scheme shown in Figure 4. Furthermore, the locally enlarged Figure 7b shows that the trigger time for switch-off current collection is 350 s prior to the transmitter bridge being switched off; this result is consistent with the set value.

#### 4.2.2. Control Timing Test

A TEM signal collection test was performed at a field experiment site in a suburb of Changchun, China. The transmitter coil used in this test was a 100 m × 100 m single-turn square coil, and the receiver coil was a circular coil with an effective area of approximate 6.2 m${}^{2}$ (radius is 0.3 m, and the number of turns is 22). In this test, the clamp voltage and matching resistance were 60 V and 50 Ω, respectively, and the transmitter-coil resistance was 0.8 Ω. When the transmission voltage was 12 V, the transmission frequency was 3.125 Hz, and the number of stacks was 32. In the low-frequency mode, the receiver coil had a resistance of 22.1 Ω and an inductance of 30.59 mH. Additionally, the amplification factor of the signal processing circuit was nine.

**Figure 8:**
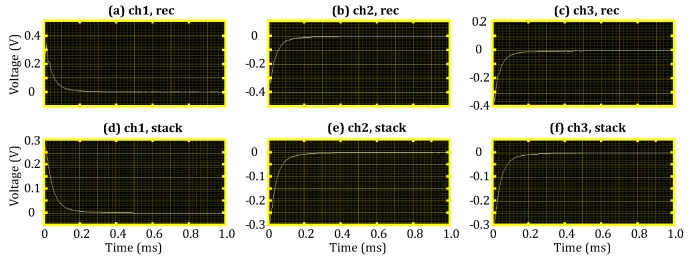
Test results for three channels of the TEM receiver. (**a**) Individual measurement curve for Channel 1; (**b**) individual measurement curve for Channel 2; (**c**) individual measurement curve for Channel 3; (**d**) stacked measurement curve for Channel 1; (**e**) stacked measurement curve for Channel 2; (**f**) stacked measurement curve for Channel 3.

The TEM receiver was designed to have multiple receiving channels; the data received by three of these channels are shown in Figure 8. Figure 8a–c shows the TEM attenuation curves obtained from an individual measurement; note that the sign of the signal is related to the winding direction of the receiver coil. Although noise is present in the individual signal measurement, as can be seen in Figure 8d–f, a smooth attenuation curve was achieved after 32 repeated TEM signal measurements were performed. In addition, the TEM signals of the three channels shown in Figure 8 are similar.

#### 4.2.3. TEM Transmission Switch-Off Current Test

**Figure 9:**
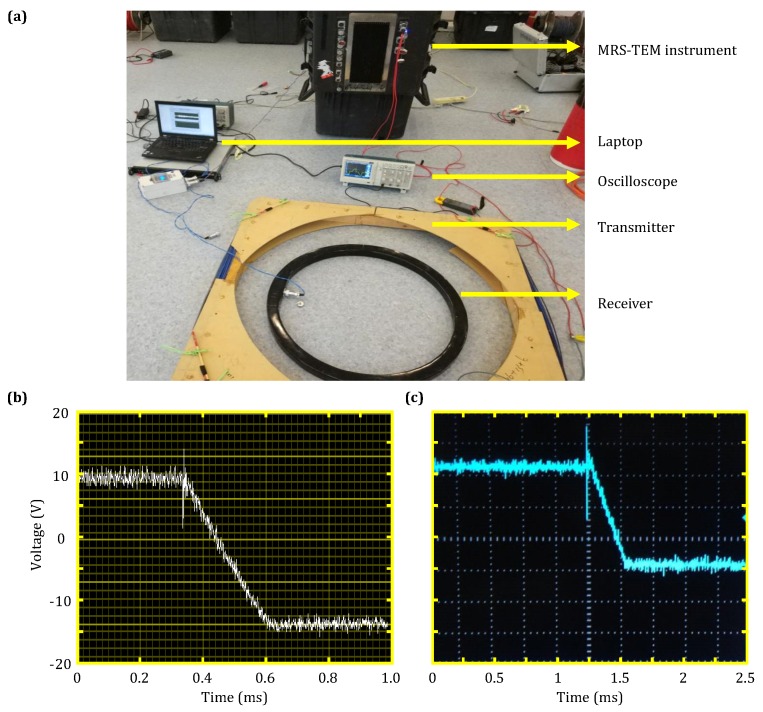
TEM transmitter switch-off current test results. (**a**) Photograph of experimental setup; (**b**) measurement results from the control module of the combined instrument experiment; (**c**) measurement results from the current clamp experiment.

A photograph of the experimental setup for the TEM transmission switch-off current test, in addition to the corresponding results, is shown in Figure 9. The TEM transmitter coil employed in this test was 1 m × 1 m, had 128 turns, a resistance of 4.3 Ω, an inductance of 43.2 mH and a transmission voltage of 10 V. The transmission clamp voltage and matching resistance was 300 V and 1000 Ω, respectively.

First, the effectiveness of the control module of the combined instrument was evaluated. The transmission current signal was converted to a voltage signal via the Hall sensor in the TEM bridge module before being transferred to the transmitter control module. The control module then transferred the collected data to the host computer for display (Figure 9b).

Then, a current clamp and oscilloscope combination was implemented and tested. The current clamp had a measuring range of 4 A and a scale ratio of 100 mV/A. The signals collected via the current clamp were displayed as voltage signals on the oscilloscope; the resulting display is shown in Figure 9c.

By converting and comparing the data shown in Figure 9b,c, it was found that both measurement methods yielded the same results: a transmission current amplitude of 2 A and a switch-off time of 280 μs.

### 4.3. Advanced Detection Test for the Combined Instrument

In order to verify the feasibility of the MRS-TEM combined detector, an advanced detection and joint interpretation test was performed in a tunnel near Qi Yue Mountain, Hubei, China. The tunnel had an axial azimuth of 319${}^{\circ }$, a length of 630 m and a maximum buried depth of 143 m. The rocks surrounding the tunnel had a relatively low strength and were prone to water-inrush and water-gushing disasters. The rocks surrounding the tunnel were mainly middle Triassic Badong Formation (T${}_{2}$b) mudstone mixed with argillaceous limestone, upper Triassic Xujiahe Formation (T${}_{3}$xj) sandstone, coal seam at the bottom of shale, lower Jurassic Zhenzhongchong Formation (J${}_{1}$z) argillaceous siltstone and mudstone mixed with sandstone. The groundwater surrounding the tunnel was mainly fissure water and loose pore water that was primarily replenished via atmospheric rainfall and primarily drained as a result of direct runoff, evaporation and drainage to rivers or slopes via small-fissure springs.

**Figure 10:**
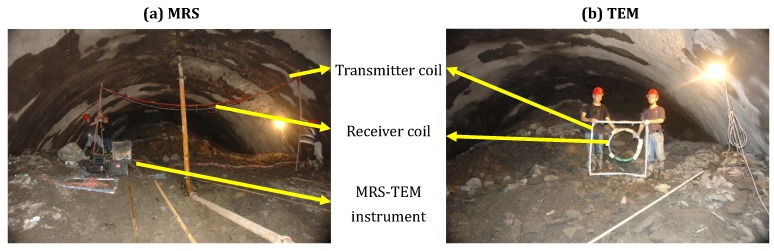
MRS-TEM instrument application site. (**a**) MRS system measurement process; (**b**) TEM system measurement process.

The combined MRS-TEM instrument was used to perform advanced detection at tunnel location GK5 + 287.5, as is shown in Figure 10. Separate transmitter and receiver coils with dimensions of 9 m × 3 m were implemented in the MRS system; the transmitter coil had eight turns, and the receiver coil had 32 turns (Figure 10a). The intensity of the local geomagnetic field was 42,300 nT, and the corresponding Larmor frequency was 1801 Hz. The transmission pulse moment had a range of 0.1–4.0 As and was set to pulse 16 times. For each pulse moment, 36 data measurements were performed in repetition. After performing data processing techniques such as spike removal, industrial frequency resonance wave removal and stacking, large ambient noise was found to contribute an average noise level of 70 nV.

A center loop method was implemented for the TEM system, with a 128-turn 1-m × 1-m square coil and 6-m${}^{2}$ air-cored coil being employed as the transmitter coil and receiver coil, respectively (Figure 10b). In this tunnel, we used a line synchronization method to synchronize the transmitter and receiver. The transmission voltage was 10 V, and the transmission frequency was 12.5 Hz. Six measurement points were selected at the cross-section of the tunnel face, with 1024 data measurements performed at each point. Because the initial bar reinforcement induced polar mutation points and jumping points in the measurement data, additional data processing methods that were not previously employed, such as smoothing and jumping point removal, were applied.

**Figure 11:**
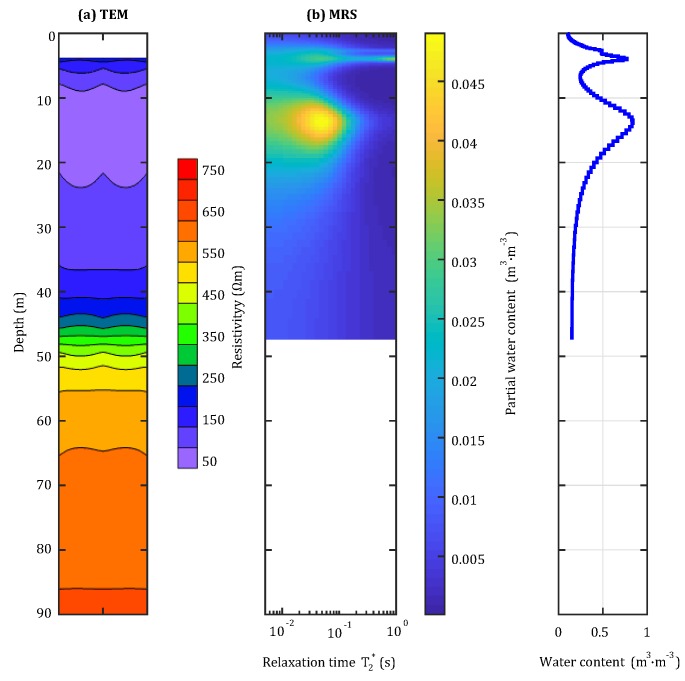
Combined system detection results.

The water content distribution and resistivity distribution up to 45 m and 90 m beyond the tunnel face, respectively, were measured by using the MRS-TEM joint inversion method described in Section 2, as is shown in Figure 11. The partial water content (PWC) obtained via MRS indicated the presence of two major aquifer areas. A maximum water content of 76% was measured from the first area, which was located 4 m within the tunnel. Additionally, because the occurrence medium was observed to have a relatively large pore size, it was concluded to be broken gravel. The second area, which was located 13.4 m within the tunnel, was found to have a maximum water content of 87%. This area was concluded to be clay because the occurrence medium was observed to have a relatively small pore size. Note that, because of the existence of shallow-depth blind areas, the TEM system could only measure the resistivity distribution of depths more than 5 m beyond the tunnel face. Analysis of the TEM system measurements revealed that the resistivity at 7–20 m was extremely low; this finding is consistent with what was expected from the location of the second aquifer area. Further analysis of the MRS-TEM joint inversion measurements revealed the distribution of areas with a relatively large water content at 2–20 m beyond the tunnel face. Through excavation, it was verified that the surrounding rocks within 20 m beyond the tunnel face had low strength and a large water content and were determined to be V-level surrounding rocks. These findings are consistent with the joint interpretation result.

## 5. Conclusions

In this study, a combined MRS-TEM detection instrument and its interpretation method were developed for construction applications in narrow underground spaces such as tunnels. Based on the common resistivity parameter, a combined interpretation of the data obtained via both methods was achieved by establishing an accurate MRS kernel function. In order to reduce the size and weight of the detection instrument so as to facilitate transportation and measurement, a combined MRS-TEM instrument was designed, and instrument control, transmission and synchronization were achieved through wireless communication. Moreover, the built-in TEM switch-off current acquisition function of the instrument provides a reliable reference for field adjustment of instrument parameters. Additionally, the effectiveness and reliability of the MRS-TEM instrument were verified via laboratory and field tests, and the practicability of the proposed instrument was verified by employing it in an actual tunnel. However, in order to improve the detection accuracy of water-induced disasters, it is necessary to further develop a multi-channel data collection module, a noise canceling module for high-noise environments and a high-resolution interpretation method.
